# The diagnostic targeting of a carbohydrate virulence factor from *M.Tuberculosis*

**DOI:** 10.1038/srep10281

**Published:** 2015-05-15

**Authors:** Conrad E. Chan, Sebastian Götze, Geok T. Seah, Peter H. Seeberger, Nestan Tukvadze, Markus R. Wenk, Brendon J. Hanson, Paul A. MacAry

**Affiliations:** 1 Department of Microbiology, National University of Singapore; 2 Max Planck Institute of Colloids and Interfaces, Department of Biomolecular Systems, Germany; 3 Department of Chemistry and Biochemistry, Freie Universität Berlin; 4 Immunology Programme, Centre for Life Sciences, National University of Singapore; 5 National Centre for Tuberculosis and Lung Diseases, Tbilisi, Georgia; 6 Department of Biochemistry, National University of Singapore; 7 Defense Medical and Environmental Research Institute, DSO National Laboratories, Singapore

## Abstract

The current clinical management of TB is complicated by the lack of suitable diagnostic tests that can be employed in infrastructure and resource poor regions. The mannose-capped form of lipoarabinomannan (ManLAM) is unique to the surface envelope of slow-growing, *pathogenic* mycobacteria such as *M.tuberculosis (M.tb)* and facilitates passive invasion of mononuclear phagocytes. The detection of this virulence factor in urine, sputum and serum has engendered interest in its employment as a biomarker for *M.tb* infection. In this study, we utilize a subtractive screening methodology to engineer the first high affinity recombinant antibody (My2F12) with exquisite specificity for the α1-2 mannose linkages enriched in ManLAM from *M.tb*. My2F12 binds to pathogenic mycobacterial species but not fast growing non-pathogenic species. Testing on matched urine and serum samples from TB patients indicates that My2F12 works in patient cohorts missed by other diagnostic methodologies.

The World Health Organisation estimates that there were 8.6 million new incident cases and 1.3 million deaths due to tuberculosis (TB) in 2012, occurring primarily in the developing countries of Africa and Asia^1^. While active TB is a treatable disease with an 85% success rate for non-drug resistant pulmonary infections, the management of TB in endemic regions is hampered by a lack of *suitable* diagnostic methodologies^2^. Current approaches include sputum culture, nucleic acid amplification tests (NAATs) or smear microscopy. Sputum culture is the gold standard diagnostic assay for pulmonary TB but requires up to two weeks for a definitive result^2^. NAATS, which have near equivalent sensitivity to sputum culture, have high costs that limit their broader employment in the developing regions where TB is most prevalent^3^. The most widely utilized diagnostic test relies upon the microscopic observation of stained mycolic acid on the surface of acid-fast bacilli in sputum samples collected from patients then smeared onto glass slides-smear microscopy. While rapid, the sensitivity of this assay has been reported to range from 20% to 80% and is highly operator dependent^4^. It also has reduced sensitivity in HIV positive cohorts and cannot distinguish between different mycobacterial species-in particular those that are pathogenic versus those that are non-pathogenic^5^.

Antibody-based detection of TB–specific biomarkers can form the basis of an inexpensive point-of-care test that has the required specificity and sensitivity. One suitable biomarker is the polysaccharide α1-2 mannose capping motif of lipoarabinomannan (Man-LAM), a membrane glycolipid reportedly found in the blood, sputum and urine of TB patients^6^,^7^,^8^. Urinary LAM in particular has been explored extensively in recent years as a basis for TB diagnosis due to its ease of collection and processing^9^. Our targeting of the mannose capping motif reduces the likelihood of false positives based on the ubiquitous expression of the LAM backbone molecule on the waxy outer-coat of all mycobacterial species^10^-the clinical utility of assays targeting the ubiquitous forms of LAM remain unproven due to their reported low sensitivity in comparison with the current methods described above, especially in HIV negative cohorts which comprise the majority of TB patients globally^7^,^9^. In particular, three separate studies have shown that such assays cannot detect smear-negative patients, a group that currently requires either NAATs or culture for detection and would benefit most from a rapid point-of-care diagnostic^7^,^11^,^12^. However, there is clear evidence that LAM can be detected in the serum and urine of individuals co-infected with HIV^8^,^13^.

In this study, we have adapted an antibody-phage display library for directed epitope targeting by prior negative depletion of pan-LAM specific antibodies to isolate the first α1-2 mannose (ManLAM) specific antibody (My2F12) for diagnostic use. We describe the characterization, molecular engineering and application of this antibody for the detection of slow growing, pathogenic mycobacteria. We also describe a methodology for enhancing the detection of α1-2 mannose caps in patients serum by prior depletion of endogenous antibodies that the inhibit binding of My2F12 We also describe a methodology for enhancing the detection of α1-2 mannose caps in patients’ serum by prior depletion and denaturation of endogenous antibodies that inhibit the binding of My2F12. Testing on a pulmonary TB HIV-negative patient cohort indicates that My2F12 can be used to detect both smear-positive and negative TB patients with high specificity in serum and urine. Thus, this antibody represents a specific reagent that can be employed for the development of a new point of care test for TB.

## Results

### Isolation of Man-LAM (mannose cap) specific antibodies by phage antibody display

**Figure 1:**
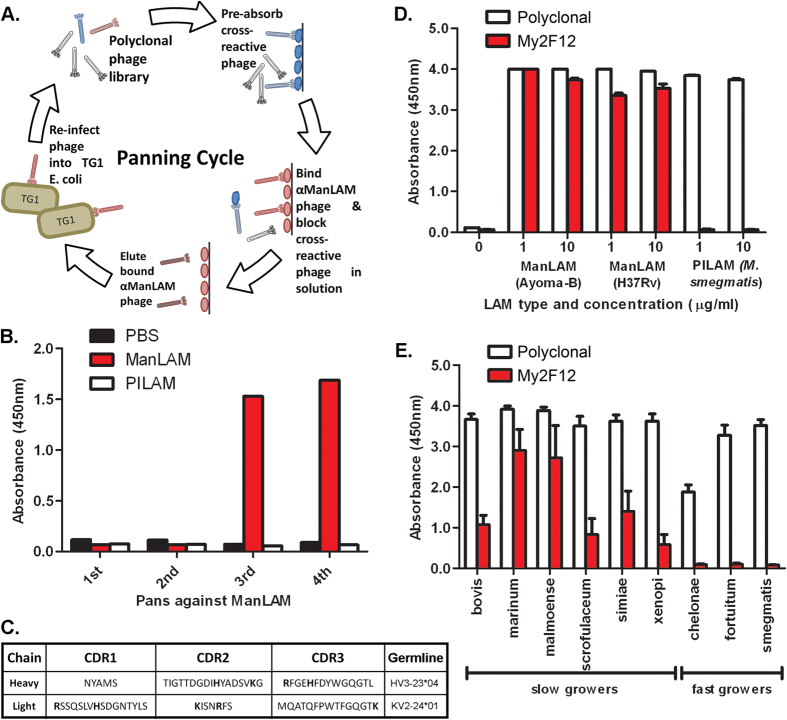
Selection of ManLAM specific antibodies. **(A)** Procedure for antibody phage panning combined with negative selection. **(B)** Selective enrichment of Humanyx library for ManLAM-specific binders as indicated by indirect polyclonal phage ELISA. **(C)** CDR amino acid sequence of clone My2F12. Basic amino acids are in bold. Sandwich ELISA using My2F12 or with commercial pan-LAM specific polyclonal on purified ManLAM and PILAM **(D)** or LAM in culture supernatant of various fast or slow-growing mycobacteria **(E).** Sandwich ELISA data are the average of three independent experiments and error bars show standard error of mean

As the mannose caps comprise only a small proportion of the entire Man-LAM molecule, we employed a related phosphoinositol-capped lipoarabinomannan (PILAM), to deplete antibodies against epitopes common to all LAM species from a non-immune human antibody phage display library to direct selection towards the cap (Fig. 1A). ManLAM-specific enrichment of the polyclonal phage library was achieved as shown by the increase in ManLAM-specific ELISA signal (Fig. 1B). No concurrent increase in binding for PILAM was observed indicating that there was no enrichment of antibodies against the common LAM backbone.

Analysis of the antibody repertoire of the enriched Pan 4 library by restriction fragment length polymorphism analysis and sequencing of selected monoclonal Fab-phages indicated that that there was only one unique clone (My2F12) present, the CDR sequence and germline variable gene source is shown (Fig. 1C). This was expressed as a human G1 antibody and then tested for ManLAM specificity by indirect ELISA. While a commercial rabbit pan-LAM specific polyclonal antibody bound to all species of LAM; the IgG1-My2F12 retained the specificity of the original Fab-phage construct and was able to distinguish ManLAM of two different *M.tb* strains from PILAM of *M. smegmatis* (Fig. 1D).

My2F12 was subsequently tested by sandwich ELISA on LAM secreted into mycobacterial culture supernatants to verify that its specificity for the mannose caps conferred the ability to distinguish LAM derived from either fast or slow growing mycobacteria. For comparison, the supernatants were also assayed on a commercial diagnostic LAM sandwich ELISA (Clearview) that relies on a pan-LAM specific rabbit polyclonal. Binding of My2F12 was only evident following incubation with supernatants from slow-growing species (Fig. 1E). In contrast, binding of the pan-LAM polyclonal was observed in the supernatants from all mycobacterial species.

**Figure 2:**
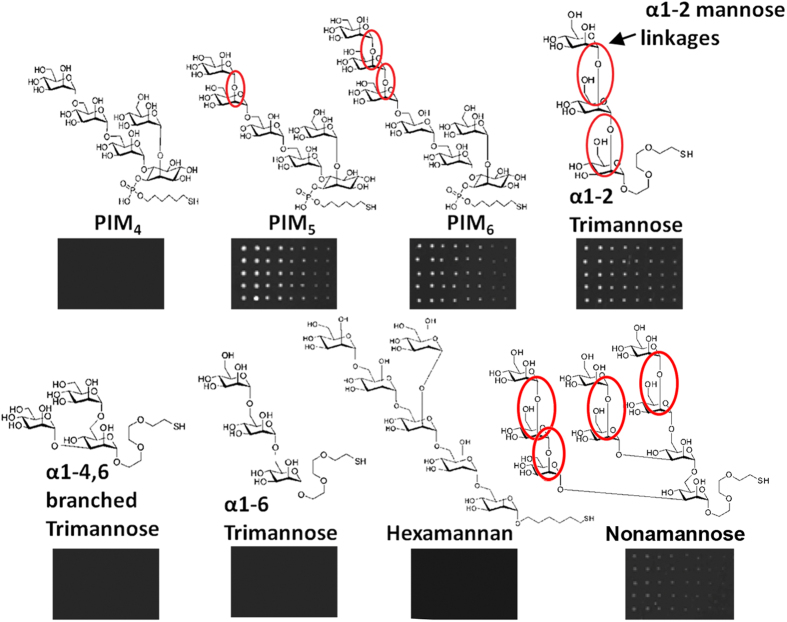
Epitope specificity of My2F12 antibody. Carbohydrate microarray showing specific binding to oligosaccharides containing terminal linear α1-2 mannose linkages (red circles) printed onto slides at decreasing concentrations (left to right).

In order to identify the types of carbohydrates motifs recognized by My2F12 we tested for antibody binding to synthetic carbohydrates printed onto microarray slides^14^. The antibody was shown to bind only mannose oligosaccharides containing the terminal linear α1-2 mannose linkages and not mannose oligosaccharides or phosphoinositol mannosides (PIMs) with only α1-4 or α1-6 mannose linkages or the branched α1-2, 6 linkage on hexamannan (Fig. 2). Binding was lost on inhibition with soluble ManLAM, but not PILAM or α1-4 linked mannan, confirming that this is the binding epitope on ManLAM. No binding was observed for other polysaccharides consisting of hexose or pentose monomers, such as maltotriose, fucose, rhamnose, lactose, galactose, and di- & hexa-arabinan (Supplementary Fig. S1), α1-2 linked mannans is the sole type of linkage found in the cap of ManLAM^9^.

### Engineering chimeric My2F12 antibodies for candidate diagnostic assays

**Figure 3:**
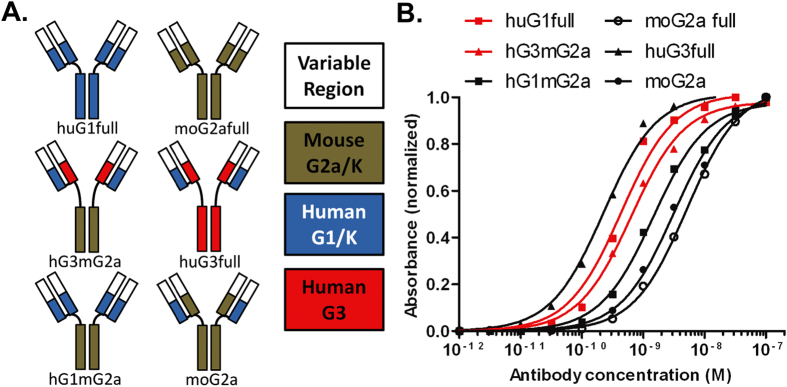
Design and avidity of chimeric antibodies. **(A)** Construction of mouse-human chimeric antibodies indicating isotype and species origin of CH1, Fc and variable regions. **(B)** Serial dilution of IgG against ManLAM on indirect ELISA showing relative affinities of different chimeric constructs. Red lines indicate parental antibody (huG1full) and engineered diagnostic (hG3mG2a)

Human antibodies are not ideal for diagnostic purposes as these are likely to give significant background staining when used on human samples. A chimeric antibody consisting of a human light chain and heavy variable region and a mouse G2a constant region would be more suitable. However, a chimeric antibody often has significantly reduced affinity relative to the parental antibody (huG1full) as modification of constant regions and antibody isotype is known to affect avidity^15^. We reasoned that a chimeric My2F12 consisting of the CH1 heavy chain constant domain and hinge of human IgG3 followed by the Fc of mouse IgG2a (hG3mG2a) would have near parental avidity, as the increased flexibility of the G3 hinge would offset any loss associated with the switch to mouse Fc (Fig. 3A). In addition a set of constructs was engineered to highlight the regions of the antibody that were important for retention of parental avidity; hG1mG2a contained the CH1 and hinge of human IgG1 coupled to the Fc region of mouse IgG2a; and two more traditional chimeric antibodies which had the complete human heavy chain constant region replaced with that of mouse IgG2a, with a fully human or chimeric light chain, moG2a and moG2afull respectively. A fully human IgG3 version (huG3full) of My2F12 was also produced.

The relative avidities of these antibodies were analyzed by serial dilution to a fixed concentration of ManLAM by indirect ELISA and plotting the logarithmic dilution curves (Fig. 3B). Both moG2a and moG2afull displayed significant reductions in avidity with a greater than one-log shift in the dilution curve relative to huG1full. As was hypothesized, hG3mG2a retained near parental avidity, as the dilution curve of hG3mG2a showed little deviation from that of the huG1full. That this retention of avidity was largely due the presence of the IgG3 hinge is supported by the observed half log reduction in the avidity of hG1mG2a which only differs in this region and that huG3full has higher than parental avidity. Interestingly, the loss of avidity between the antibody pairs huG1full and hG1mG2a, along with huG3full and hG3mG2a was similar, suggesting that the effects of switching hinge and Fc on avidity are additive and that despite increased flexibility of the hinge, it does not completely isolate the Fab region from changes in the Fc structure.

### Identification of pathogenic mycobacteria with My2F12

**Figure 4:**
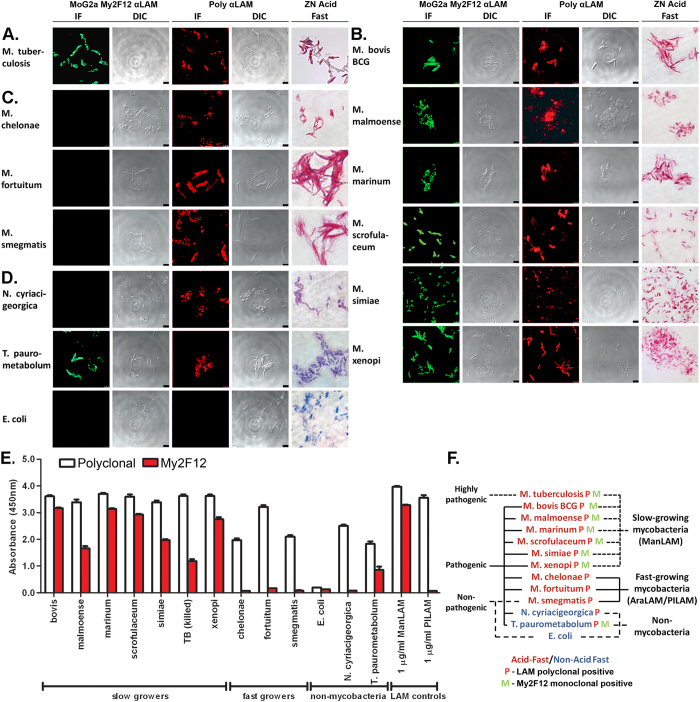
Bacterial specificity of My2F12 chimeric antibody. **(A–D)** Confocal immunofluorescence microscopy (IF) with My2F12 chimeric antibody or pan-LAM polyclonal with corresponding differential interference contrast (DIC) microscopy images and Ziehl-Neelsen acid-fast staining for fixed heat-killed *M. tuberculosis* **(A)** and fixed live cultures of slow-growing mycobacteria **(B)**, fast-growing mycobacteria **(C)** and non-mycobacterial **(D)** species. **(E)** Sandwich ELISA with hG3mG2a detector and huG1full as capture or with commercial ELISA using pan-LAM specific polyclonal against whole bacteria. My2F12 is specific only for mycobacterial slow-growers and a unique actinomycete *T. paurometabolum* while the polyclonal binds all mycobacterial and actinomycete species tested. Data are the average of three independent experiments and error bars show standard error of mean **(F)** Tree diagram showing classification of mycobacterial and non-mycobacterial species and their pathogenicity, antibody specificity and acid-fast staining.

For diagnostic employment, chimeric My2F12 antibodies must detect pathogenic mycobacteria in clinically relevant settings. One example is immunofluorescence (IF) to enhance specificity and sensitivity on sputum smears^16^. We therefore evaluated the specificity of My2F12 for diverse species of mycobacteria and actinomycetes via confocal IF microscopy. For comparison, IF with a pan-LAM specific polyclonal and the standard Ziehl–Neelsen acid-fast stain was carried out. We included two non-mycobacterial actinomycete species that had previously been isolated from clinical sputum samples and cross-reacted with polyclonal pan-LAM antibodies-these have the potential to give false positives; *Nocardia cyriacigeorgica* and *Tsukamurella paurometabolum*^7^. The Gram negative bacterium *Escherichia coli* was included as a negative control. A classification of the species used, their pathogenicity, IF and acid-fast stain results is shown in Fig. 4F.

My2F12 showed clear fluorescent staining of heat-killed *M. tuberculosis* bacilli fixed by methanol–acetone onto slides (Fig. 4A). This suggests that our antibody has the potential to detect tuberculosis infection via IF sputum smears. *M. tuberculosis* and other slow-growing bacilli also stained positively with the pan-LAM polyclonal and the acid-fast stain (Figs. 4A,4B). However, in contrast to My2F12, these also uniformly stained fast growing, non-pathogenic mycobacterial strains such as *M.smegmatis* (Fig. 4C). All non-mycobacterial actinomycete species stained negative with the acid-fast dye and My2F12 but positive with the pan-LAM polyclonal antibody with the exception of *T. paurometabolum* (Fig. 4D). To further determine the likelihood of additional cross-reactivity with associated false positive results, we tested My2F12 against common throat-resident bacterial species such as *Streptococcus*, *Staphylococcus* and *Pseudomonas aeruginosa* using the same IF methodology- no significant staining was observed (Supplementary Fig. S2).

Another possible clinical assay is the sandwich ELISA, which has an advantage over sputum smear microscopy in that it does not require a trained technician for slide preparation and analysis. We therefore tested our My2F12 chimeric antibody alongside the pan-LAM antibody sandwich ELISA (Clearview) using intact whole bacteria. For this assay, the antibody showed identical specificity to that obtained with confocal IF microscopy. Binding was evident only with the slow-growing mycobacteria and *T. paurometabolum* (Fig. 4E). In contrast, the pan-LAM polyclonal antibody bound all mycobacterial and actinomycete species. Critically, *M. tuberculosis* gave a clear signal on the My2F12 ELISA further indicating its utility as a diagnostic reagent.

### Detection of ManLAM mannose caps in sera

**Figure 5:**
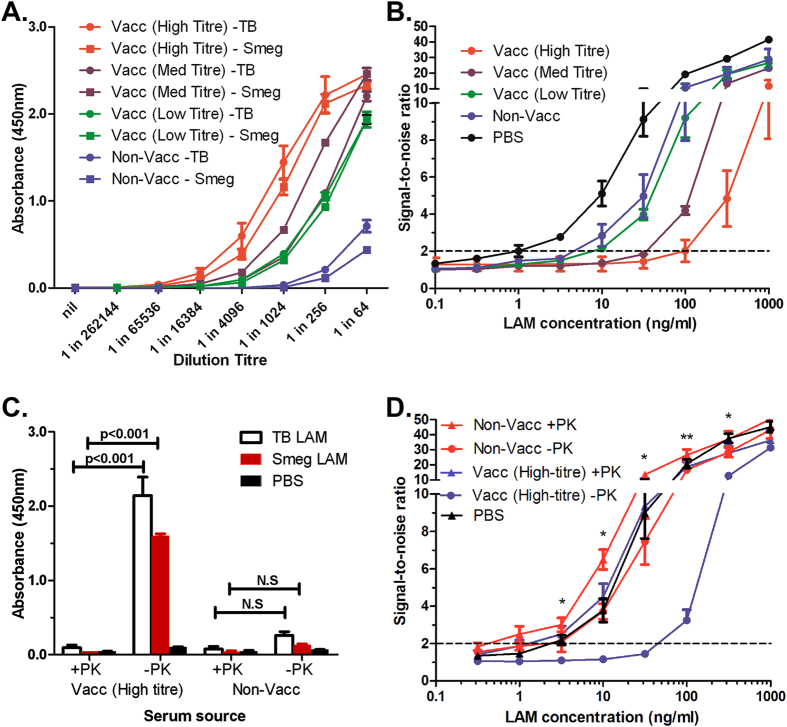
Improvement of My2F12 assay sensitivity by serum denaturation. **(A)** Anti-LAM antibody titres from BCG vaccinated and non-vaccinated individuals. **(B)** Sandwich ELISA signal-to-noise ratio showing inverse correlation between levels of endogenous anti-LAM antibodies and limit of detection. A limit of detection (dotted line) is set at a ratio of 2 and noise is defined as the signal obtained with no LAM added **(C)** Significant reduction of levels of endogenous anti-LAM antibodies in serum (at 1:512 dilution) by Proteinase K and heat treatment. **(D)** Improvement in sensitivity of sandwich ELISA as after denaturation of endogenous anti-LAM antibodies in serum samples with proteinase K and heat treatment (+PK) as compared to untreated spiked serum (-PK) and PBS standards. All data shown are the average of three independent experiments and error bars show standard error of mean. Concentrations of ManLAM are that in whole blood spiked before coagulation. (* p < 0.05, ** p < 0.01, significant difference between +PK and –PK in high-titre serum).

The potential for cross-reactivity and associated false-positive results in sputum, combined with the challenges inherent with microscope based quantification suggest that a new type of detection assay based on alternative clinical material is required. The LAM backbone has been identified in sera and urine^6^,^8^. However, endogenous LAM/anti-LAM immune complexes complicate the urinary detection of LAM and these may also confound serum based detection assays of Man-LAM^17^,^18^. To determine if this is a factor, we evaluated sandwich ELISA assay sensitivity in spiked sera from donors vaccinated with BCG in comparison with a non-vaccinated individual. The sera were first assayed for antibodies against ManLAM and PILAM by indirect ELISA. As expected, BCG vaccinated individuals exhibited higher levels of anti-LAM antibodies and these were classified into high, medium and low titres respectively (Fig. 5A). When spiked with ManLAM and assayed, serum sensitivity correlated with the level of endogenous anti-LAM antibodies (Fig. 5B). In the highest titre serum the limit of detection was approximately twenty-five-fold less than in the non-vaccinated serum controls (100 ng/ml vs 4 ng/ml) and a hundred-fold less than in LAM spiked into PBS (100 ng/ml vs 1 ng/ml), suggesting that circulating anti-LAM antibodies can confound Man-LAM detection in serum.

To address this, we developed a hybrid sample-processing methodology that first employs proteinase K to digest and precipitate serum proteins that can then be removed by low speed centrifugation. This is followed by heating the samples to 95 °C to denature any remaining antibodies and proteinase K. Indirect ELISA against PILAM and ManLAM confirmed that this procedure destroyed endogenous antibodies, as the binding activity present in the high titre serum was reduced to below that of the unvaccinated serum and near background (Fig. 5C). The procedure was then repeated with ManLAM spiked serum and assayed by sandwich ELISA in comparison to untreated serum. Treatment of both the high-titre and unvaccinated serum as described resulted in a marked improvement in the limit of detection, from 3 ng/ml to 0.5 ng/ml in the unvaccinated serum and from 50 ng/ml to 2 ng/ml in high-titre serum (Fig. 5D). In both cases, sensitivity in the treated serum was similar to that of ManLAM spiked into PBS. In addition, we also tested the same treatment process on whole blood to see if coagulation to recover serum was unnecessary. To ensure full digestion, incubation with Proteinase K was extended for 30 min. However, the limit of detection was 6 ng/ml for both high-titre and unvaccinated serum, representing an improvement for the high-titre sample but a decrease for the unvaccinated serum-this compares poorly with both the PBS standard and treated serum (Supplementary Fig. S3). These results suggest that proteinase K and heat treatment can be used to eliminate endogenous anti-LAM antibodies, thus allowing unimpeded detection of ManLAM in sera with My2F12.

### Employment of My2F12 for detection of Man-LAM in TB patient serum and urine

Previous studies employing polyclonal antibodies that target the LAM backbone molecule have suggested that LAM is higher in the sera and urine of HIV positive individuals. The key challenge remains the detection of Man-LAM in HIV negative individuals who comprise the majority of TB patients^13^. Processed urine and serum samples were tested from four separate patient cohorts (TB-negative, smear-negative & culture positive, smear grade 1+ and smear grade 2+ and above) using the sample processing and ELISA methodology detailed above with optimized antibody concentrations (Supplementary Fig. S4). Optimal cut-off values were determined based on receiver operating characteristic (ROC) plots of sensitivity and false positive rates for each type of clinical specimen with an emphasis on obtaining low false positive rates (<10%) (Supplementary Fig. S5). With these cut off values, sensitivity and false positive rates could be determined for both TB-negative and –positive cohorts. For testing of individual samples, sensitivity ranged from 26.6%–46.7% for urine and 26.6%–33.3% for serum with a false positive rate of 6.7%–7.7% (Table 1). Sensitivity was higher in the smear-positive cohort compared to the smear-negative cohort for both sample types but this was not statistically significant. The sensitivity rate was significantly higher than the false positive rate for only the smear-positive serum samples. When the absorbance values of serum and urine were combined and a new cut-off determined, all three TB-positive cohorts had statistically significant higher sensitivities above the false positive rate from 46.7%–66.6% (Table 1). The trend for higher sensitivities for the smear-positive cohorts remained but was not statistically significant.

## Discussion

We have developed an antibody with exquisite specificity for the mannose-capped form of LAM produced by slow growing, pathogenic mycobacteria, My2F12. The major pathogenic species of mycobacteria include *M. tuberculosis*, *avium*, *bovis* and *leprae;* and all fall within this group of slow-growers. Thus, our antibody is likely to be useful for detecting infections arising from such pathogens; even in the presence of non-pathogenic fast growing commensal species such as *M. smegmatis*. We have also demonstrated that our antibody is specific for the α1-2 mannose linkages that form the major carbohydrate structure of the capping motifs in Man-LAM.

While phage display libraries have been used to generate anti-carbohydrate antibodies, this is the first time a subtractive depletion technique has been applied to focus antibody specificity to a particular antigenic region on a carbohydrate molecule^19^. As the utility of diagnostic antibodies depends on their specificity, this methodology for generating highly specific, high-affinity anti-carbohydrate antibodies represents a new modus for the production of diagnostic candidates. Typically, panning of phage libraries produces multiple antibodies specific for the target antigen^19^,^20^. In this case, all isolated antibodies from our enriched library were clones of one unique high-affinity antibody, indicating a limited set of ManLAM-specific antibodies within our library or that competition for a limited antigen resulted in only one high-affinity antibody displacing all other low-affinity clones during enrichment. Either scenario suggests that this antibody specificity can be classed as rare.

The clinical utility of our antibody is dependent on whether it can be used in techniques such as IF microscopy or ELISA to detect the relevant pathogens and complement the other clinical criteria that are used as part of a diagnosis. Previous studies have shown that similar to sputum smear microscopy, the shortcomings of other anti-LAM antibodies are that they cannot distinguish between pathogenic and non-pathogenic mycobacterial species and that they cross-react with other LAM producing actinomycete species such as *Nocardia*. Despite their high-affinity for the LAM backbone molecule this potentially limits their clinical value^7^,^21^. My2F12 does not cross-react with fast growing mycobacterial species such as *M. smegmatis*, *chelonae* and *fortuitum* and was able to detect all slow-growing mycobacterial species tested using both confocal IF microscopy and ELISA.

The division of mycobacterial species into these two taxonomical groups was initially based on the observed difference in growth-rates, but was later supported by phylogenetic analysis of 16 s ribosomal RNA^22^. While the vast majority of slow growers are pathogenic in humans or animals, the majority of fast growers are environmental and non-pathogenic with a small number of exceptions that cause mild disease^22^,^23^. Our observation that ManLAM capping motifs were present in all slow growing species but none of the fast-growing suggests that these motifs may be considered as one of the defining characteristics of this group. This is a key finding, as many slow-growing species such as *M. malmonese*, *marinum*, *scrofulaceum*, *simiae* and *xenopi* have not had their LAM structure previously characterized. Moreover, My2F12 binds to a related actinomycete, *Tsukamurella paurometabolum*, which is probably due the fact that it expresses LAM with terminal α1-2 mannose linkages, the exact epitope recognized by My2F12^24^.

While the reactivity of My2F12 to all slow-growing mycobacteria could result in false positive detection of non-tuberculous mycobacteria (NTM) infection, the majority of these species e.g. *M. marinum* or *M. ulcerans* cause infections that are non-pulmonary in nature e.g. cutaneous or disseminated infection and can easily be distinguished on the basis of clinical symptomology^23^. Pulmonary NTM infection is typically caused by only a select few species such as *M. avium* or *M. kansasii*, which cannot be clearly distinguished by currently used diagnostic methods such as smear microscopy and culture. In such cases, detection can only be confirmed by *M.tb* specific molecular biology or antigen detection assays^23^,^25^. The purpose of our assay then would be to act as a replacement for sputum smears and a triage test to eliminate non-slow growing mycobacterial infections and also allow for rapid initiation of containment measures and therapy for severe infections prior to confirmation by *M.tb-*specific assays. In developing world countries where *M.tb* infections are prevalent, treatment can be initiated without need for confirmation of species as NTM infections are rare and usually found in HIV infected and immunocompromised individuals or those with a history of lung disease^25^. In our study, we did not find NTM infection in any patient, confirming the rarity of its occurrence, but also prevented us from evaluating any potential cross-reactivity in diagnostic samples.

For diagnostic employment, we engineered a form of My2F12 with a murine Fc that retains parental avidity by switching to a human G3 hinge, thus enabling detection with anti-mouse reagents and reducing background staining in human clinical samples. The minimal background binding observed in our human serum ELISA validates this approach (Fig. 5B,D). While changes in affinity and specificity have been reported due to a switch in antibody isotype or species origin^15^, no general observation for an increase in avidity by switching to G3 from G1 has been made. Our findings suggest a simple but effective method for improving the avidity of recombinant diagnostic antibodies without loss of specificity by use of a human G3 hinge.

The utility of LAM as a biomarker for TB has not been conclusively established although a significant amount of work has been carried out on urine. Unfortunately, the majority of studies indicate that urinary LAM immunoassays on their own are not significantly more sensitive than sputum smears, with the exception of the HIV positive cohort^7^,^10^,^11^,^13^,^26^. One possible explanation for this is that in immunocompetent individuals, LAM is retained in the circulation by formation of immune complexes with anti-LAM antibodies^17^. Alternative, the use of pan-LAM antibodies in earlier assays may have resulted in higher background in the TB-negative population making it harder to differentiate the true positives. Also, a previous study on sputum indicated low specificity due to the cross reactivity of polyclonal anti-LAM antibodies with other microbial flora^7^. As our antibody has fine specificity for Man-LAM, it represents a better basis for detection with this type of clinical sample. Alternatively, testing serum avoids complications linked to sputum as blood is more likely to be sterile. Previous studies showed good specificity for LAM but poor sensitivity, which was attributed to interference from endogenous anti-LAM antibodies^8^,^18^. As part of this study, we have developed a simple method for removal of these antibodies to improve sensitivity.

Testing of My2F12 on urine samples from TB patients and controls indicates a significant improvement compared to that observed in previous HIV-negative cohorts. Three separate studies had shown that sensitivity with polyclonal pan-LAM specific antibodies was 0% in the smear-negative cohort and 9-23.5% in smear-positive cohort^7^,^11^,^12^. In contrast, we obtained 26.6% and 40-46.7% in the same cohorts respectively and reached higher sensitivities of up to 66.7% with a combination of serum and urine samples (Table 1). This may be due to the reduced level of background staining provided by the increased specificity of our antibody. The potential of higher background staining in assays relying on pan-LAM specific antibodies has been demonstrated in a recent study where concentration of urine samples for higher sensitivity also resulted in higher signals in the TB-negative cohort^12^. In our assay, we also observed a small proportion of false-positives in the TB-negative cohort; this could potentially be due to LAM released from latent TB infections, which have no microbiological or radiological indications. Such infection is difficult to detect as it requires the use of immune response assays such as Quantiferon or Tuberculin Skin Test which are slow and required specialized training to perform^27^. Given that latent TB requires different treatment from active TB and is not contagiousness, it is probably more crucial for our TB diagnostic to be able to first distinguish active TB.

Most importantly, our assay was able to detect up to half of the entire smear-negative cohort, which is the group of patients who would benefit most from a cheap point-of-care test as current detection is restricted to either culture or NAATs. This performance is close to that achieved by the WHO-endorsed Xpert MTB/RIF NAAT^28^. Given that Man-LAM levels are expected to be even higher in the HIV-positive cohort, our assay should provide enhanced sensitivity compared to the pan-LAM ELISAs currently employed^17^. As new immunodiagnostics techniques with sensitivity approaching the single molecule level have been developed, such assays could also be combined with our ManLAM specific antibodies^29^. Such assays are more likely to have improved sensitivity and specificity when used with My2F12 compared to pan-LAM specific antibodies due to the reduced background. We have therefore highlighted the benefits of a ManLAM specific antibody for the detection of TB and demonstrated its potential in urine and serum clinical specimens with an emphasis on those that are currently missed by standard methodologies. This combination of urine and serum testing represents a novel approach to the detection of TB and could prove useful where highly sensitive but resource-intensive assays are not available, particularly in developing regions where TB is prevalent.

## Methods

### Ethics statement

Serum was obtained from healthy adult Singaporean volunteers with informed consent under National University Hospital Domain Specific Review Board Protocol No. 08-171 while clinical samples (serum, urine, sputum) were obtained from adult Georgian volunteers with informed written consent after review by the National Centre for Tuberculosis and Lung Diseases Institutional Ethics Committee. All experiments were carried out in accordance with approved guidelines.

### Collection of clinical samples and study design

Adult volunteers were recruited from patients presenting with symptoms suggestive of pulmonary TB infection (persistent fever, night sweats, cough of more than 2 weeks duration, haemoptysis, chest pain, loss of weight and/or appetite, and generalized fatigue) at the National Centre of Tuberculosis and Lung Diseases, Tbilisi, Georgia according to the following inclusion criteria: >18 years of age, HIV negative, no recent illness or hospitalization within the last two weeks. Matched sputum, serum and urine samples were collected prior to initiation of treatment and stored at -80 °C prior to processing. Sputum samples were used concurrently for clinical diagnosis via light microscopy acid-fast sputum smear and culture per standard WHO diagnostic criteria and the presence of *M.tb* rather than non-tuberculous mycobacteria confirmed by Genotype line probe assay (HAIN lifesciences, Germany). Patients were then classified into four groups based on the diagnostic results: non-TB (confirmed microbiologically negative by sputum smear microscopy and culture and without any clinical suspicion of TB), TB smear-negative (smear microscopy-negative but culture positive), TB smear grade 1+ only (10–100 acid-fast bacilli/100 microscopy fields) and TB smear grade 2+ (>100 acid-fast bacilli/100 microscopy fields) and above. 15 patients were recruited from each group. The average age, sex ratio and clinical symptoms of each group is given in Supplementary Table S1. No significant difference between the groups was found in any of these parameters. Patients diagnosed only on the basis of clinical presentation and radiology without any microbiological confirmation of TB were excluded from this study. Treatment for TB infected patients was subsequently carried out as per standard WHO DOTS (Directly Observed Therapy-Short Course) protocols. Serum was denatured for 2 hrs at 55 °C. Approximately 5 ml of mid-stream urine was collected and incubated in a plastic sample container at 95 °C for 30 min in a drying oven. Samples were then allowed to cool to room temperature and stored at −20 °C before testing by ELISA.

### Phage display panning and screening of monoclonal phage antibodies

A non-immune human Fab-antibody phage display library (Humanyx Pte Ltd, Singapore) was screened for anti-LAM antibodies after multiple rounds of selection on ManLAM (Nacalai Tesque, Japan) coupled with prior negative depletion on *M. smegmatis* PILAM (Invivogen, US)^30^. Briefly, cross-reactive antibodies were first depleted from the library by preadsorption to 100 μg PILAM coated onto a Maxisorb immunotube (Nunc, Denmark). Remaining cross-reactive antibodies were blocked in solution by the addition of 100 μg of PILAM. The phage library was then applied to an immunotube coated with 100 μg of ManLAM to isolate anti-ManLAM antibodies. Non-specific phage was then removed by repeated washing, recovered with trypsin digestion and a fresh enriched library produced according to standard panning procedures^31^. Panning was carried out for four rounds with pre-absorption for the first two rounds.

### Engineering Human-Mouse chimeric antibodies

Human G1 and G3 CH1 sequences and Mouse G2a CH2 and CH3 (Fc) sequences were amplified with primers designed to permit a second overlapping PCR of the human CH1 and mouse Fc sequences to obtain chimeric antibodies. (See Supplementary Materials and Methods for detailed protocol) Antibodies were then expressed and purified as previously described except for G3 antibodies which were purified on Protein G^31^.

### ELISA methodologies

All ELISA assays included a casein blocking step and incubations for 1 hr at room temperature with 100 ul sample volume per well with four PBS washes between incubations, unless indicated. Colour was developed by incubation with TMB and stopped with sulphuric acid. For indirect ELISAs, 96-well Maxisorb ELISA plates (Nunc, Denmark) were coated with LAM antigen (sourced as above, H37Rv LAM was provided by Colorado State University) in PBS overnight at 4 °C. ELISA plates were then washed twice with PBS and blocked with 380 μl per well of Casein block (Thermo Scientific, US) at room temperature for 2 hrs. The plates were washed twice and 1:10 polyclonal phage, 1 μg/ml purified My2F12 or HRP-conjugated pan-LAM specific rabbit polyclonal from the Clearview kit (Inverness Medical Innovations, UK) was added. Plates were washed and phage and antibody detected with 1:5000 HRP-conjugated anti-M13 (GE Healthcare, US) or anti-human Fc polyclonal antibody (Thermo Scientific, US) as appropriate.

For sandwich ELISA, plates were coated with 5 μg/ml capture antibody in 1xPBS overnight at 4 °C, blocked and washed as above before adding antigen. Live mycobacterial culture was grown to log-phase in Middlebrook 7H9 media (BD Diagnostics, US) at 37 °C (with 5% CO_2_ for BCG). *Mycobacterium smegmatis* was provided by Dr Ayi Teck Choon, DSO National Laboratories. *Nocardia cyriacigeorgica* (ATCC BAA-1517) and *Tsukamurella paurometabolum* (ATCC 8368) was purchased from American Type Culture Collection, USA and cultured as per manufacturer’s protocol. Culture was spun down to separate intact bacteria and supernatant; the pellet was resuspended in PBS to OD 0.5 and the supernatant filter-sterilized separately. *M.tb* (H37Rv strain) was inactivated at 90 °C for 30 min (provided by Dr Sylvie Alonso). After antigen binding, the plate was washed and 5 μg/ml detector antibody added, washed again and 1:5000 HRP-conjugated anti-mouse Fc polyclonal antibody (Thermo Scientific, US) added. Sandwich ELISA with pan-LAM specific polyclonal was carried out using pre-coated plates and HRP-conjugated antibody from Clearview commercial LAM ELISA kit (Inverness Medical Innovations, UK) according to manufacturer’s instructions. For serial dilution ELISAs of My2F12 IgG to plot avidity curves, plates were prepared with LAM as described for indirect ELISA. Antibodies were added and detected with either anti-human or mouse Fc-HRP as appropriate at the same dilutions as above, but all incubations were carried out at 37 °C to ensure binding had reached equilibrium. (See Supplementary Methods for ELISA optimization protocol)

### Immunofluorescence (IF) and acid fast bacilli staining

10^6^ cfu of mycobacteria was fixed onto a cover slip with 500 μl of 1:1 v/v of methanol-acetone for 30 min at -20 °C for IF or 5% phenol-70% ethanol solution for 15 min at room temperature for acid fast staining respectively and air-dried. For IF, fixed cover slips were blocked with 2% fetal calf serum in 1xPBS, washed with PBS and then incubated with either 5 μg/ml My2F12 chimeric antibody or 1:200 pan-LAM specific rabbit polyclonal (Acris Antibodies, Germany) in blocking solution. Cover slips were then washed in PBS/Tween, incubated with secondary antibody, either anti-rabbit Alexa 594 or anti-mouse polyclonal Alex 488 (Invitrogen, US) respectively, at 1:100 dilution in blocking solution, before mounting with Mowiol. Mounted slides were viewed using a Confocal Laser Microscope system (Leica, Germany). Incubations were for 30 min at room temperature. For acid fast staining, fixed slides were stained with TB Quickstain (BD Diagnostics, US) according to manufacturer’s instructions in 24-well plates. Cover slips were allowed to dry and then fixed to glass slides and viewed under a light microscope (Leica, Germany). Throat bacterial smears were processed in the same manner (see Supplementary Methods for detailed protocol)

### Carbohydrate microarrays

Carbohydrates were synthesized according to previously published procedures^14^. (See Supplementary Methods for detailed protocol)

### Denaturation of serum antibodies

In serum spiking experiments, Man-LAM was added to whole blood before coagulation and then the blood was allowed to clot at room temperature and serum harvested by centrifugation at 2000 g for 15 min. Spiked serum or whole blood was treated with 1mg/ml final concentration of Proteinase K (Sigma-Aldrich, US) for 1hr or 1.5 hr respectively at 55 °C, then centrifuged at 2000 g for 15 min to pellet precipitate and then heated for 30 min at 95 °C. Untreated serum underwent standard heat inactivation for 1 hr at 55 °C. To test for endogenous anti-LAM antibodies, Maxisorb plates (Nunc, Denmark) were coated with 10 μg/ml LAM or PBS and blocked as described for indirect ELISAs and then serum diluted in casein block was applied. Sandwich ELISAs were carried out as described above using huG1full as capture and hG3mG2a as detector and specific absorbance determined by subtracting the absorbance from the PBS control.

### Data analysis and statistics

The raw absorbance values of the three TB-positive cohorts and one TB-negative control cohort were used to plot an ROC curve of sensitivity versus false positive rates (1-specificity) as shown in Figure S5, with the three TB-positive groups treated as a single cohort. 95% confidence levels for specificity and sensitivity were also determined from the ROC analysis. For data analysis using a combination of matched urine and serum sample data, a sum of the two absorbance values were used. Tests for significance were carried out using 1 way ANOVA with Bonferroni corrections for multiple T-test comparisons or Chi-square tests for continuous and categorical data sets respectively while affinity binding curves were plotted using non-linear regression with a single-binding site specific binding model. All statistical analysis was carried out using Prism 5.0 software (GraphPad Software, United States). Test for significant differences between sensitivity and false positive percentages were carried out using multiple Z-test for independent groups ( http://www.mccallum-layton.co.uk/stats/ZTestTwoTailSampleSize.aspx).

## Author Contributions

C.E.C. and S.G. performed the experiments; C.E.C., S.G., P.H.S., B.J.H. and P.A.M. designed the experiments; G.T.S., N.T. and M.R.W. provided reagents, patient samples and other assistance; C.E.C., B.J.H. and P.A.M. wrote the paper.

## Additional Information

**How to cite this article**: Chan, C. E. *et al.* The diagnostic targeting of a carbohydrate virulence factor from *M.Tuberculosis*. *Sci. Rep.* **5**, 10281; doi: 10.1038/srep10281 (2015).

## Supplementary Material

Supporting Information

## Figures and Tables

**Table 1:** LAM ELISA performance in various patient groups and specimen types.

**Patient group**	**TB -ve Specificity (95% CI)**	**TB +ve Sensitivity (95% CI)**		
		**Sm -ve**	**1+**	**>1+**
**Sample Type**				
Serum	**93.3%**(68.1-99.8%,n = 15)	**26.7%**(7.8-55.1%,n = 15)	**20.0%**(4.3-48.1%,n = 15)	**33.3%**(68.1-99.8%,n = 15)
Urine	**92.3%**(64.0-99.8%,n = 13)	**26.7%**(7.8-55.1%,n = 15)	**46.7%**(21.3-73.4%,n = 15)	**40.0%**(16.3-67.7%,n = 15)
Serum + Urine	**92.3%**(64.0-99.8%,n = 13)	**46.7%**(21.3-73.4%,n = 15)	**66.7%**(38.4-88.2%,n = 15)	**66.7%**(38.4-88.2%,n = 15)

Sensitivity in urine, serum and a combination of both samples are shown for individual TB-positive cohorts (Sm-ve: smear-negative, culture positive; 1+: smear grade 1+ only; >1+: smear grade 2+ and above). Specificity (1- false positive rate) is given for the TB-negative (TB -ve) cohort. Number of samples per cohort (n) is indicated along with the 95% confidence level limits (95% CI)

## References

[b1] Global *Tuberculosis Report 2013*. (World Health Organization, 2013).

[b2] Keeler E. *et al.* Reducing the global burden of tuberculosis: the contribution of improved diagnostics. Nature 444 **Suppl 1**, 49–57 (2006). 17159894 10.1038/nature05446

[b3] Kambashi B. *et al.* Utility of nucleic acid amplification techniques for the diagnosis of pulmonary tuberculosis in sub-Saharan Africa. Int. J Tuberc Lung Dis. 5, 364–369 (2001). 11334256

[b4] Steingart K. R. *et al.* Sputum processing methods to improve the sensitivity of smear microscopy for tuberculosis: a systematic review. Lancet Infect. Dis. 6, 664–674 (2006). 17008175 10.1016/S1473-3099(06)70602-8

[b5] Elliott A. M. *et al.* Negative sputum smear results in HIV-positive patients with pulmonary tuberculosis in Lusaka, Zambia. Tuber Lung Dis. 74, 191–194 (1993). 8369514 10.1016/0962-8479(93)90010-U

[b6] Boehme C. *et al.* Detection of mycobacterial lipoarabinomannan with an antigen-capture ELISA in unprocessed urine of Tanzanian patients with suspected tuberculosis. Trans. R Soc. Trop. Med. Hyg. 99, 893–900, (2005). 16139316 10.1016/j.trstmh.2005.04.014

[b7] Dheda K. *et al.* Clinical utility of a commercial LAM-ELISA assay for TB diagnosis in HIV-infected patients using urine and sputum samples. PLoS One 5, e9848 (2010). 20352098 10.1371/journal.pone.0009848 PMC2844421

[b8] Sada E., Aguilar D., Torres M. & Herrera T. Detection of lipoarabinomannan as a diagnostic test for tuberculosis. J Clin Microbiol. 30, 2415–2418 (1992). 1401008 10.1128/jcm.30.9.2415-2418.1992 PMC265515

[b9] Minion J. *et al.* Diagnosing tuberculosis with urine lipoarabinomannan: systematic review and meta-analysis. Eur. Respir. J 38, 1398–1405 (2011). 21700601 10.1183/09031936.00025711

[b10] Nigou J., Gilleron M. & Puzo G. Lipoarabinomannans: from structure to biosynthesis. Biochimie 85, 153–166 (2003). 12765785 10.1016/s0300-9084(03)00048-8

[b11] Reither K. *et al.* Low sensitivity of a urine LAM-ELISA in the diagnosis of pulmonary tuberculosis. BMC Infect. Dis. 9, 141 (2009). 19715562 10.1186/1471-2334-9-141 PMC2741465

[b12] Savolainen L. *et al.* Modification of clearview tuberculosis (TB) enzyme-linked immunosorbent assay for TB patients not infected with HIV. Clin Vaccine Immunol. 20, 1479–1482 (2013). 23825194 10.1128/CVI.00375-13 PMC3889579

[b13] Talbot E. *et al.* Test characteristics of urinary lipoarabinomannan and predictors of mortality among hospitalized HIV-infected tuberculosis suspects in Tanzania. PLoS One 7, e32876 (2012). 22412939 10.1371/journal.pone.0032876 PMC3297608

[b14] Horlacher T. & Seeberger P. H. Carbohydrate arrays as tools for research and diagnostics. Chem. Soc. Rev. 37, 1414–1422 (2008). 18568167 10.1039/b708016f

[b15] McCloskey N., Turner M. W., Steffner P., Owens R. & Goldblatt D. Human constant regions influence the antibody binding characteristics of mouse-human chimeric IgG subclasses. Immunology 88, 169–173 (1996). 8690447 10.1111/j.1365-2567.1996.tb00001.x PMC1456430

[b16] Orholm M., Holten-Andersen W. & Lundgren J. D. Improved detection of Pneumocystis carinii by an immunofluorescence technique using monoclonal antibodies. Eur. J Clin Microbiol. Infect. Dis. 9, 880–885 (1990). 2073898 10.1007/BF01967503

[b17] Wood R. *et al.* Lipoarabinomannan in urine during tuberculosis treatment: association with host and pathogen factors and mycobacteriuria. BMC Infect. Dis. 12, 47 (2012). 22369353 10.1186/1471-2334-12-47 PMC3349560

[b18] Sarkar S. *et al.* A bispecific antibody based assay shows potential for detecting tuberculosis in resource constrained laboratory settings. PLoS One 7, e32340 (2012). 22363820 10.1371/journal.pone.0032340 PMC3283739

[b19] Schoonbroodt S. *et al.* Engineering antibody heavy chain CDR3 to create a phage display Fab library rich in antibodies that bind charged carbohydrates. J Immunol. 181, 6213–6221 (2008). 18941211 10.4049/jimmunol.181.9.6213

[b20] Mao S. *et al.* Phage-display library selection of high-affinity human single-chain antibodies to tumor-associated carbohydrate antigens sialyl Lewisx and Lewisx. Proc. Natl. Acad. Sci. USA 96, 6953–6958 (1999). 10359820 10.1073/pnas.96.12.6953 PMC22023

[b21] Kaur D., Lowary T. L., Vissa V. D., Crick D. C. & Brennan P. J. Characterization of the epitope of anti-lipoarabinomannan antibodies as the terminal hexaarabinofuranosyl motif of mycobacterial arabinans. Microbiology 148, 3049–3057 (2002). 12368438 10.1099/00221287-148-10-3049

[b22] Shinnick T. M. & Good R. C. Mycobacterial taxonomy. Eur. J Clin Microbiol. Infect Dis. 13, 884–901 (1994). 7698114 10.1007/BF02111489

[b23] Griffith D. E. *et al.* An official ATS/IDSA statement: diagnosis, treatment, and prevention of nontuberculous mycobacterial diseases. Am J Respir. Crit. Care Med. 175, 367–416 (2007). 17277290 10.1164/rccm.200604-571ST

[b24] Gibson K. J. *et al.* Tsukamurella paurometabola lipoglycan, a new lipoarabinomannan variant with pro-inflammatory activity. J Biol. Chem. 279, 22973–22982 (2004). 15031299 10.1074/jbc.M310906200

[b25] McGrath E. E., McCabe J., and Anderson P. B., Guidelines on the diagnosis and treatment of pulmonary non-tuberculous mycobacteria infection. Int. J Clin Pract., 62, 1947–55. (2008). 19166441 10.1111/j.1742-1241.2008.01891.x

[b26] Flores L. L. *et al.* Systematic review and meta-analysis of antigen detection tests for the diagnosis of tuberculosis. Clin Vaccine Immunol. 18, 1616–1627 (2011). 21832100 10.1128/CVI.05205-11 PMC3187027

[b27] Guidelines on the management of latent tuberculosis infection . (World Health Organization, 2015). 25973515

[b28] Theron G. *et al.* Evaluation of the Xpert MTB/RIF assay for the diagnosis of pulmonary tuberculosis in a high HIV prevalence setting. Am J Respir. Crit. Care Med. 184, 132–140 (2011). 21493734 10.1164/rccm.201101-0056OC

[b29] Schmidt R. *et al.* Single-molecule detection on a protein-array assay platform for the exposure of a tuberculosis antigen. J Proteome. Res. 10, 1316–1322, (2011). 21247063 10.1021/pr101070j

[b30] de Haard H. J. *et al.* A large non-immunized human Fab fragment phage library that permits rapid isolation and kinetic analysis of high affinity antibodies. J Biol. Chem. 274, 18218–18230 (1999). 10373423 10.1074/jbc.274.26.18218

[b31] Lim A. P. *et al.* Neutralizing human monoclonal antibody against H5N1 influenza HA selected from a Fab-phage display library. Virol. J 5, 130, (2008). 18957074 10.1186/1743-422X-5-130 PMC2582236

