# Supplementary material for: The diagnostic targeting of a carbohydrate virulence factor from M.Tuberculosis
Source: Sci Rep. 2015 May 15;5:10281. doi: 10.1038/srep10281 (PMC4432570; doi:10.1038/srep10281)
Supplement: Supporting Information [file srep10281-s1.pdf]

Supplementary Information

The diagnostic targeting of a carbohydrate virulence factor from *M.Tuberculosis*.

Authors: Conrad E. Chan, Sebastian Götze, Geok T. Seah, Peter H. Seeberger, Nestan Tukvadze, Markus R. Wenk, Brendon J. Hanson, Paul A. MacAry

Supplementary methods

Carbohydrate microarrays

In brief, GAPS II slides (Corning, US) were submerged in DMF containing 2% diisopropylethlyamine and 0.5 mg/mL maleimido-N-hydroxysuccinimide hexanoic acid for 14 h at room temperature. Functionalized slides were washed three times with methanol, dried and stored under an argon atmosphere. The functionalized oligosaccharides were synthesized as described previously (1-6). The oligosaccharide compounds with one molar equivalent TCEP to reduce disulfides were dissolved to the desired concentration in PBS. Per spot, 1 nL of the solution was printed onto the maleimide functionalized slides using an automated printing robot in replicates of ten at four different concentrations (2 mM, 0.4 mM, 80 µM and 16 µM). Slides were incubated in a humid chamber to complete reaction for 24 h and stored in a dessicator until usage. Printed carbohydrate microarray slides were washed three times with water and quenched in 0.1 % (v/v) β-mercaptoethanol in PBS for 1 h at room temperature. Afterwards, slides were washed three times with water and two times with ethanol. Slides were blocked by a solution of 2.5% BSA in Tris buffer with calcium (20mM Tris, 150 mM NaCl, 1 mM $CaCl_2$, pH 7.4 for 1 h at room temperature, washed three times with Tris buffer with calcium and centrifuged. The antibody (50µg/mL) was incubated on the slides in in 20mM HEPES, 150 mM NaCl, 1 mM $CaCl_2$, pH 7.4, washed three times with the same Buffer and

centrifuged. For inhibition, mannan, PILAM or ManLAM (each at a final concentration of 30 µg/mL) was added to the incubation solution. A fluorescence-labeled detection antibody (10 µg/mL in 20mM HEPES, 150 mM NaCl, 1 mM $CaCl_2$, pH 7.4; Invitrogen) was incubated on the slide in a similar fashion. Afterwards, slides were scanned using a fluorescence microarray scanner (GenePix 4300A, Molecular Devices).

Primer sequences and PCR protocols for construction of Human-Mouse chimeric antibodies.

Human G1 CH1: SEQ1B 5'-CGGATCTCTAGCGAATTCC-3' & HuG1MoG2aRev 5'-CCACCCAAGAGGTTAGGTGCTGGGCACGGTGGGCATGTG-3'; human G3 CH1: SEQ1B and HuG3MoG2aRev 5'-CCACCCAAGAGGTTAGGTGCTGGGCACCGTGGG CATGGGGG-3'; Mouse G2a: HuG1MoG2aFor 5'-CACATGCCCACCGTGCCCAGCAC CTAACCTCTTGGGTGG-3' or HuG3MoG2aFor 5'-CCCCCATGCCCACGGTGCCCAG CACCTAACCTCTTGGGTGG-3' with MoG2aRev 5'-ATCCAGCTTCTAGACTATTTACC CGGAGTCCGGGAGAAG-3'. PCR was carried out for 30 cycles at an annealing temperature of 56°C for 30s and extension at 72°C for 1min for CH1 sequences and an annealing at 62°C for 30s and extension at 72°C for 2min for Fc sequences. A second PCR to overlap the CH1 and Fc sequences was done with five cycles of PCR without primers at an annealing temperature of 62°C for 30s and extension at 72°C for 2min, followed by 25 rounds of PCR with SEQ1B and MoG2aRev primers added at an annealing temperature of 56°C for 30s to amplify the final chimeric sequence which was then cloned into the heavy chain expression vector described below.

Immunofluorescence microscopy of common throat bacterial cultures

Common throat bacterial smears provided by Dr Timothy Barkham, Tan Tock Seng Hospital. were formalin inactivated and heat fixed onto glass slides. These were then incubated with 5µg/ml My2F12 chimeric antibody in blocking buffer (5%BSA in 1x PBS) for 1hr, washed and probed with Alexa Fluor 488 anti-mouse polyclonal and washed again as above. After washing, DAPI mounting medium (Invitrogen, USA) was added and the smear covered with a glass slip and visualized at 100x power using a fluorescence microscope (Olympus BX51, USA) under UV and 488nm illumination for detection of DAPI and secondary antibody respectively.

Optimization of sandwich ELISA for clinical samples

For initial optimization of antibody concentration, serum denatured as described above was spiked with 2ng/ml of ManLAM and tested using various combinations of antibody concentrations as shown in Figure S4, in the sandwich ELISA protocol above using huG1full and hG3mG2a My2F12 variants as capture and detector respectively. The optimal concentration was selected based on the highest signal-to-noise ratio and signal-to-noise difference which was 10µg/ml capture and 5µg/ml detector antibody. At these antibody concentrations, the assay limit of detection (signal-to-noise ratio of 2) was 0.5ng/ml of ManLAM in 1xPBS. Sandwich ELISA on clinical samples was then carried out using 100µl of sample pre-mixed with 10µl of 10x PBS. TMB was incubated for 15min or 30min for urine and serum respectively for colour development.

Supplementary references

1. D. M. Ratner, E. W. Adams, J. Su, B. R. O'Keefe, M. Mrksich, P. H. Seeberger, Probing protein-carbohydrate interactions with microarrays of synthetic oligosaccharides. *Chembiochem* **5**, 379-382 (2004).
2. S. Boonyarattanakalin, X. Liu, M. Michieletti, B. Lepenies, P. H. Seeberger, Chemical synthesis of all phosphatidylinositol mannoside (PIM) glycans from Mycobacterium tuberculosis. *J Am Chem Soc* **130**, 16791-16799 (2008).
3. E. Crouch, K. Hartshorn, T. Horlacher, B. McDonald, K. Smith, T. Cafarella, B. Seaton, P. H. Seeberger, J. Head, Recognition of mannosylated ligands and influenza A virus by human surfactant protein D: contributions of an extended site and residue 343. *Biochemistry* **48**, 3335-3345 (2009).
4. A. Holemann, B. L. Stocker, P. H. Seeberger, Synthesis of a core arabinomannan oligosaccharide of Mycobacterium tuberculosis. *J Org Chem* **71**, 8071-8088 (2006).
5. M. A. Oberli, M. Tamborrini, Y. H. Tsai, D. B. Werz, T. Horlacher, A. Adibekian, D. Gauss, H. M. Moller, G. Pluschke, P. H. Seeberger, Molecular analysis of carbohydrate-antibody interactions: case study using a Bacillus anthracis tetrasaccharide. *J Am Chem Soc* **132**, 10239-10241 (2010).
6. E. W. Adams, D. M. Ratner, H. R. Bokesch, J. B. McMahon, B. R. O'Keefe, P. H. Seeberger, Oligosaccharide and glycoprotein microarrays as tools in HIV glycobiology; glycan-dependent gp120/protein interactions. *Chem Biol* **11**, 875-881 (2004).

Supplementary Tables:

Patient parameters	TB -ve (n=13)	TB +ve (n=15 per group)		
		Sm -ve	1+	>1+
Average Age	45	40	34	42
Male:Female	1.17	4.00	1.14	2.75
Clinical symptoms				
Cough	92.3%	86.7%	100.0%	93.3%
Haemoptysis	15.4%	13.3%	26.7%	26.7%
Chest pain	38.5%	33.3%	60.0%	26.7%
Fatigue	100.0%	93.3%	80.0%	93.3%
Fever	69.2%	80.0%	80.0%	86.7%
Night Sweats	61.5%	86.7%	80.0%	73.3%

Table S1. Summary of patient parameters. There was no significant difference in the frequency of clinical symptoms in each group, or in age or sex ratio as determined by chi-square test and 1-way ANOVA (for age)

Supplementary Figures:

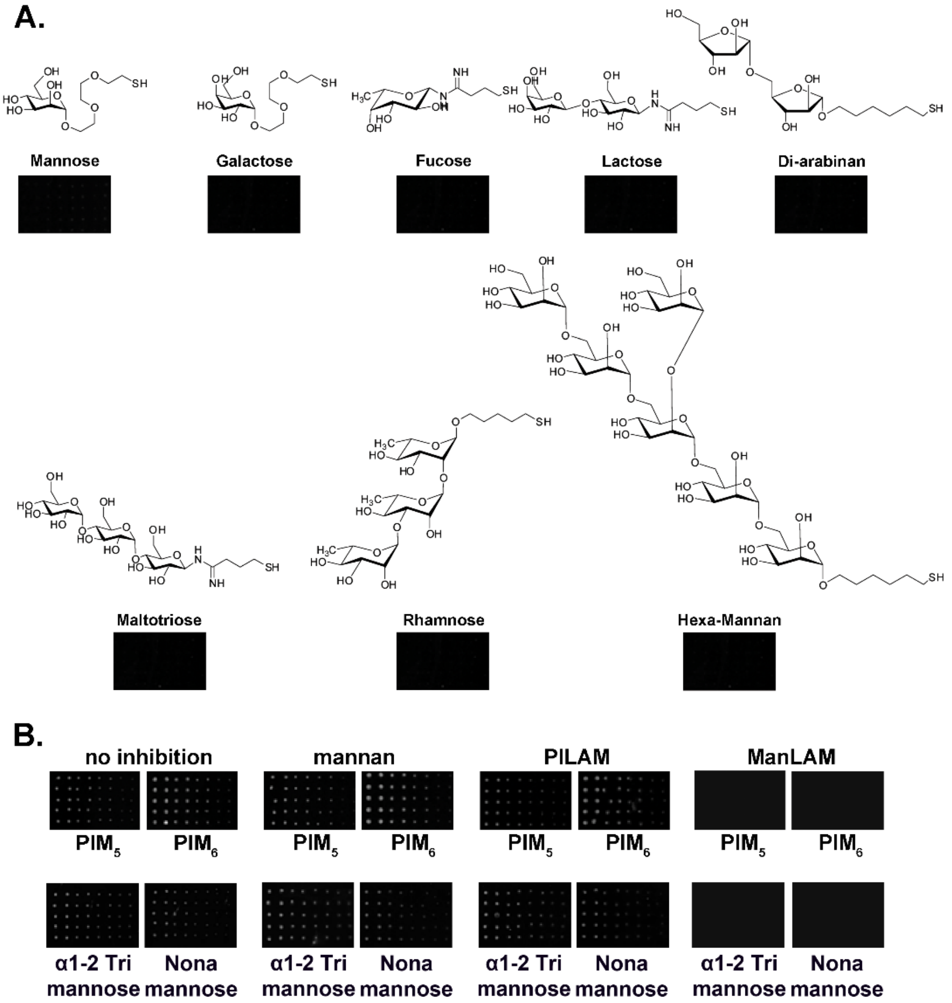

Fig. S1 α1-2 mannose specificity of My2F12. (A) Lack of antibody binding to other mono, di- and oligosaccharides as shown by carbohydrate microarray. **(B)** Loss of binding to oligomannosides PIM_5, PIM_6, α1-2 Trimannose and nonamannose by inhibition with 30µg ManLAM but not with equivalent amounts of PILAM or mannan.

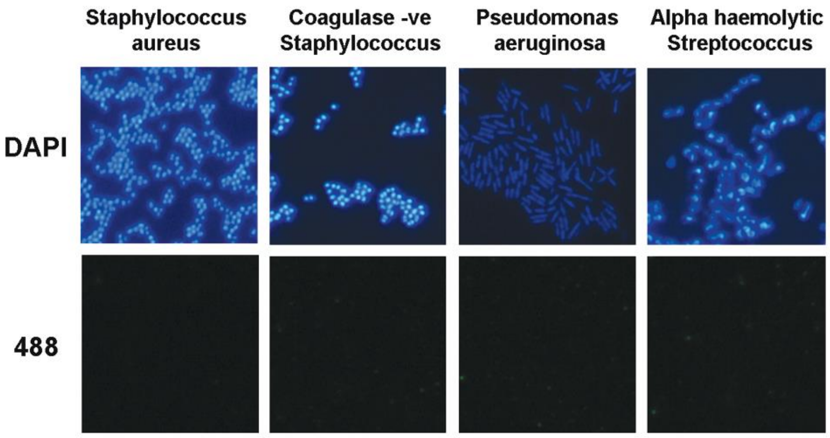

Fig. S2 My2F12 specificity for slow-growing mycobacteria. Lack of binding of My2F12 chimeric antibody to various strains of common throat bacteria as shown by immunofluorescence microscopy. Presence of actual bacilli is highlighted with the DAPI stain and the corresponding image under 488nm illumination indicates absence of secondary antibody and My2F12 binding.

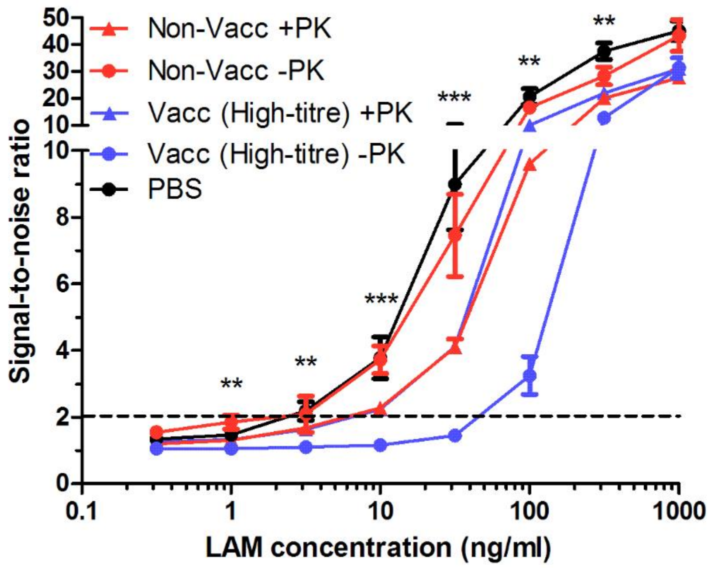

Fig. S3 Improvement in My2F12 assay sensitivity after treatment of whole blood. Changes in sensitivity of sandwich ELISA as indicated by signal-to-noise ratio after denaturation of endogenous anti-LAM antibodies in spiked whole blood with proteinase K and heat treatment (+PK) as compared to untreated spiked serum (-PK) and PBS standards. Treatment of whole blood improves sensitivity in high-titre serum but not in unvaccinated serum. All figure data are the average of three independent experiments and error bars show standard error of mean. Concentrations of ManLAM are that in whole blood spiked before coagulation. (** p<0.01, *** p<0.001, significant difference between +PK and –PK in high-titre whole blood)

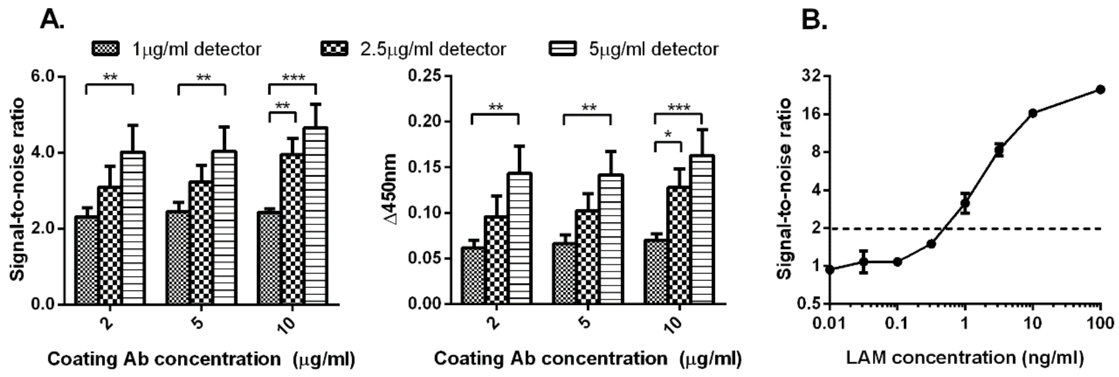

Fig. S4 Optimization of capture and detector antibody concentration. (A) Significant enhancement of signal-to-noise ratio (left panel) and specific absorbance above background (right panel) at different antibody concentrations in sandwich ELISA, using spiked serum with a low concentration of ManLAM (2ng/ml) **(B)** Limit of detection of 0.5ng/ml (at signal-to-noise ratio of 2) at optimal antibody concentrations of 10µg/ml capture and 5µg/ml detector antibody with a 100µl sample of ManLAM spiked into PBS. All results are the average of three independent experiments and the error bars show standard error of mean. *: $p<0.05$, **: $p<0.01$, ***: $p<0.001$

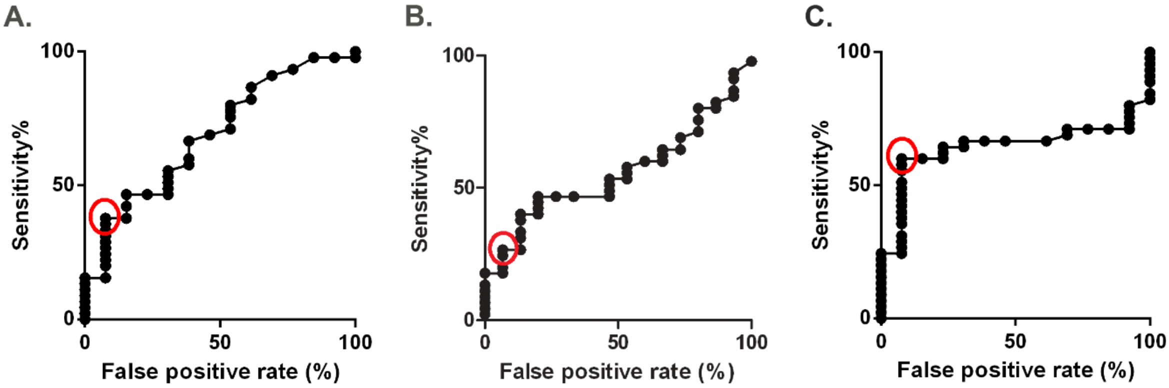

Fig. S5 Determination of cut-off values by ROC analysis. ROC chart indicating variation of false positive rate with sensitivity at increasing absorbance cut-off values for **(A)** urine **(B)** serum **(C)** urine + serum. Red circle indicates optimal point for greatest sensitivity with minimum false positive rates to obtain maximum accuracy.
